# The validity of a new resilience scale: the Japan Resilience Scale (J-RS) for mothers with a focus on cultural aspects

**DOI:** 10.1186/s12889-025-22765-6

**Published:** 2025-04-28

**Authors:** Mariko Takeuchi, Michiko Matsunaga, Akimitsu Miyake, Ryuichiro Egashira, Sayaka Hotta, Mai Nakano, Misaki Moriguchi, Fumihiko Yasuno, Masako Myowa, Keisuke Hagihara

**Affiliations:** 1 Department of Advanced Hybrid Medicine, Osaka University Graduate School of Medicine, Suita, Osaka Japan; 2 Graduate School of Education, Kyoto University, Kyoto, Japan; 3 Japan Society for the Promotion of Science, Tokyo, Japan; 4 Tohoku University School of Medicine, Sendai, Miyagi Japan; 5 Department of Psychiatry, National Center for Geriatrics and Gerontology, Obu, Aichi Japan

**Keywords:** Resilience, Japan Resilience Scale (J-RS), Center for Epidemiologic Studies Depression (CES-D), Social connection, Emotional regulation

## Abstract

**Background:**

Resilience has been recognized as a potential outcome for preserving mental health and preventing postpartum depression. Social connection and emotional regulation have been identified as possible factors in resilience from social and cultural perspectives. Therefore, it is necessary to develop a scale that is adapted to the Japanese cultural milieu.

**Objective:**

To establish the validity and reliability of the new resilience scale, questionnaires were administered to mothers raising children aged 0–5 years.

**Methods:**

The Japan Resilience Scale (J-RS) is a newly created, 25-item, self-report scale designed to assess resilience. The J-RS includes six subscales (Joy, Anger, Apprehension, Grief, Fear, and Social connection) that are rated on a 5-point Likert scale (0–4). To validate the J-RS, data were collected from 238 mothers (mean age 35.3 ± 4.7 years), and its relationships with other measures, such as the Resilience Scale (RS) and the Center for Epidemiologic Studies Depression (CES-D) scale, were examined. Internal consistency (reliability) of the J-RS was assessed using Cronbach’s alpha coefficient, and the final model was determined via confirmatory factor analysis (CFA). Multiple logistic regression analysis was then used to identify depressive symptoms using the CES-D.

**Results:**

A total of 238 mothers, with 18.5% exhibiting depressive symptoms, were analyzed. The mean J-RS score was 61.3 ± 14.2. J-RS was positively correlated with RS (*r* = 0.71, *p* < 0.01) and negatively with CES-D (*r* = -0.62, *p* < 0.01). These results demonstrate the construct validity of the J-RS. CFA confirmed the six-factor model’s good fit for emotion and social connection. The J-RS was effective in distinguishing individuals at risk of depression (AUC = 0.83, 95% CI: 0.75–0.91). At a cutoff of 59/60, sensitivity, specificity, positive predictive value, and negative predictive value were 84.1%, 63.9%, 34.6%, and 94.7% respectively.

**Conclusions:**

The J-RS appears to be the optimal index for assessing resilience, and would allow screening for postpartum depression among Japanese mothers. This study also showed that emotional control and social connectedness are important components of resilience.

**Supplementary Information:**

The online version contains supplementary material available at 10.1186/s12889-025-22765-6.

## Background

Maintaining the mental health of mothers is an important challenge. In Japan, about 10–15% of mothers experience maternal depression [1, 2], and it increased to 20–30% during the COVID- 19 pandemic [3, 4]. In addition, prolonged postpartum depression, which was previously considered to occur within the first year postpartum, is now being reported, indicating the need for longer-term follow-up. A high percentage of patients with depression in primary care report only physical symptoms [5]. Furthermore, mothers tend to avoid disclosing mental symptoms, with only approximately 30% seeking medical consultation [6]. Therefore, screening for postpartum depression is particularly challenging. In response, we have developed a new scale, the Multidimensional Physical Scale (MDPS), which can independently detect the risk of depression based on physical symptoms without the need for emotional questions, and we have validated its reliability [7]. As the next step, this study aimed to develop effective support methods using factors that help reduce depressive tendencies or improve depressive symptoms, particularly considering the current situation in which many mothers find it difficult to access medical care. Promoting resilience may play an important role in supporting the reduction and improvement of depressive tendencies. Postpartum depression arises from the interaction of physical, psychological, and social factors. To assess resilience in postpartum women from a multifaceted perspective, we consider it essential to establish a robust and reliable method.

Resilience is “the ability to maintain the persistence of one’s orientation towards existential purposes” [8]. Resilience also refers to how an individual faces stress and adversity, and low resilience has been linked to poorer outcomes [9]. The results of the analysis of 848 Japanese healthcare workers [10] and the meta-analysis of 1094 articles involving elderly populations similarly [11] showed an inverse correlation between resilience and depression. They showed that resilience is an important variable in mental health. Several reports have also implicated resilience as important in the prevention or treatment of postpartum depression [9]. Resilience is an outcome shaped by the interaction of various psychosocial protective factors, including stress coping [12], adaptability to challenges [13], social support, optimism [14], emotional regulation, and self-esteem [15]. However, the conceptual understanding of resilience remains ambiguous, and its specific components have yet to be clearly defined.

The resilience scale (RS) [16] is one of the most globally used measurements to assess the degree of individual resilience and has been successfully used in many studies. During validation in Japan, differences were found, such as lower total scores and weaker correlations with depression, and no correlations with stress compared with the original [17]. These differences might be because the traditional resilience scale focuses on individualism. The custom of valuing harmony, or empathy and social ties, is not unique to Japan, but at least in Japan, harmony with the surroundings tends to be more important than individualism. In addition, social connections are important for mothers raising children [18]. Effective maternal emotional regulation is important for adapting to parenthood [19]. Therefore, to determine which components of resilience are appropriate for Japanese mothers, we were inspired by Japanese concepts of Kampo. Japanese Kampo treatments are often used to improve physical and mental conditions, including depression, during pregnancy and child rearing [20, 21]. In Japanese Kampo medicine, the functions of the human body and mind are categorized into five systems: (i) Heart, (ii) Liver, (iii) Spleen, (iv) Kidney, and (v) Lung. Each system corresponds to a specific emotion within this framework: (i) Joy, (ii) Anger, (iii) Apprehension, (iv) Grief, and (v) Willingness [22]. In Kampo medicine, it is recognized that emotional confusion can lead to illness through its effects on the corresponding physiological functions [23]. Consequently, evaluating the current state of each of the five systems and addressing any imbalances is a fundamental treatment approach in Kampo medicine [24].

The aim of this study was to develop and validate a psychometric tool for assessing resilience of mothers, and to evaluate its effectiveness in the early screening of postpartum depression. From a psychometric perspective, we hypothesized that resilience consists of multiple dimensions, particularly emotional regulation and social connections, which are essential in the Japanese cultural context. Methodologically, the aim was to assess the scale’s reliability and validity through factor analysis and correlation with established measures.

## Methods

### Participants

The participants were 238 mothers aged between 24 and 48 (mean 35, SD 4.7) years. They were registered as volunteers at Kyoto University. All children of the participants were considered their offspring, with 58.6% being first-born. The sociodemographic and clinical characteristics of the participants are shown in Table 1a.

**Table 1:** Participants’ characteristics (a) and the J-RS score (b)

(a) Characteristics (*N* = 238)	
Age, years	35.3 ± 4.7
Time after birth, months	25.3 ± 17.4
Resumption of menstruation, yes (%)	200 (84.0)
Number of children, n	1.5 ± 0.6
Education, years	16.0 ± 2.2
Household income	
Low: Less than 3-million-yen, n (%)	10 (4.2)
Middle: 3–7-million-yen, n (%)	98 (41.2)
High: More than 7-million-yen, n (%)	123 (51.7)
No answer, n (%)	7 (2.9)
CES-D, mean ± SD (0–63)	10.2 ± 8.0
Normal (0–15), n (%)	194 (81.5)
With depressive symptoms (16–63), n (%)	44 (18.5)
RS, mean ± SD (25–175)	95.2 ± 17.2
RSES, mean ± SD (10–50)	28.8 ± 5.7
SSQ-Number, mean ± SD	4.1 ± 2.8
SSQ-Satisfaction, mean ± SD	5.0 ± 0.9
PSS, mean ± SD (0–56)	25.9 ± 6.9
SF- 8: Physical, mean ± SD (0–100)	49.1 ± 6.7
SF- 8: Mental, mean ± SD (0–100)	47.9 ± 6.6
MDPS-M, mean ± SD (0–34)	10.3 ± 5.7
(b) Characteristics (*N* = 238)	
J-RS (*N* = 238)	
J-RS, mean ± SD (0–100)	61.3 ± 14.2
J-RS Joy, mean ± SD (0–16)	9.9 ± 2.7
J-RS Anger, mean ± SD (0–16)	7.7 ± 3.0
J-RS Apprehension, mean ± SD (0–16)	7.8 ± 4.0
J-RS Grief, mean ± SD (0–16)	7.2 ± 3.7
J-RS Willingness, mean ± SD (0–16)	10.9 ± 2.9
J-RS Social connection, mean ± SD (0–20)	17.7 ± 3.1

*Abbreviations*: *J-RS* Japan Resilience Scale, *RS* Resilience Scale, *CES-D* Center for Epidemiologic Studies Depression, *SSQ* Social Support Questionnaire, *PSS* Perceived Stress Scale, *RSES* Rosenberg Self-Esteem Scale, *SD* Standard deviation

### Measures

*Socioeconomic Status (SES).* SES is assessed based on a combination of economic and social factors, such as education, income, and family structure. Income is described in three groups, low income (less than 3-million-yen), middle income (3 to 7-million-yen), and high income (more then 7-million-yen), based on three quartiles of public Japanese data [25]. All of the data are shown in Table 1a.

*Resilience Scale (RS)* [16]*.* The RS is one of the most widely used instruments for assessing individual resilience. It was originally developed by Wagnild and Young, and it has been adapted into Japanese by Nishi et al. The RS measures resilience through two factors: *personal competence* (17 items) and *acceptance of self and life* (8 items). Items are rated on a 7-point Likert scale, with total scores ranging from 25 to 175. The Japanese version of the RS demonstrated high internal consistency (Cronbach’s alpha = 0.90) and validity. In this study, the RS showed a reliability coefficient (Cronbach’s alpha) of 0.88.

*Center for Epidemiologic Studies Depression (CES-D)* [26]. The CES-D is commonly used to assess depressive symptoms and was developed by the National Institute of Mental Health and standardized for use in Japanese using the split-half method (*r* = 0.79). It consists of 20 items rated on 4-point scales (range, 0–60), with a cutoff score of 16 points indicating depression. The reliability in the sample of this study, as measured by Cronbach’s alpha, was 0.87.

*Rosenberg Self-Esteem Scale (RSES)* [27]. The RSES, originally developed by Rosenberg, is widely used to measure self-esteem, and a Japanese version was adapted by Mimura et al. (Cronbach’s alpha = 0.81) [28]. It consists of 10 items, and each item assesses the degree of self-esteem on a 5-point Likert scale, ranging from strongly agree to strongly disagree (range, 10–50). Cronbach’s alpha for reliability in the sample of this study was 0.88.

*Social Support Questionnaire (SSQ)* [29]*.* The SSQ, originally developed by Sarason et al., is a widely used tool for measuring an individual’s perceived social support and satisfaction with it. It has been used extensively in research and clinical settings. The Japanese version of the SSQ was adapted by Furukawa et al. and has been shown to have high internal consistency, factor validity, and construct validity [30]. The short version of the SSQ consists of 12 items, with six items measuring the perceived number of social supports, and the other six items measuring satisfaction with social support. Each item is rated on a 6-point Likert scale, and the average scores for the two domains are calculated.

*Perceived Stress Scale (PSS)* [31]*.* The PSS, developed by Cohen et al., measures an individual’s perception of stress in their life, and it was standardized for the Japanese population by Mimura et al. [32]. The Japanese version of the PSS has high internal consistency (Cronbach’s alpha = 0.74) and factor validity. It consists of 14 items that ask about how often a person has experienced feelings of stress in the past month, and the degree of stress for each item is rated on a 5-point Likert scale (range, 0–56). In this study’s sample, reliability, as indicated by Cronbach’s alpha, was 0.77.

*Short Form Health Survey (SF- 8)* [33]. The SF- 8 is a widely used 8-item instrument of health-related quality of life (QOL) that assesses eight domains of physical (SF- 8 Physical) and mental (SF- 8 Mental) health functioning. Scores range from 0 to 100, with higher scores representing better health.

*Multidimensional Physical Scale for Mothers (MDPS-M)* [7]*.* The MDPS-M is a simple, self-reported, screening scale for depression of women involved in child-rearing from physical aspects, developed by Takeuchi et al. The MDPS-M consists of 17 questions and resumption of menstruation. The psychometric properties indicate good internal consistency among Japanese mothers (Cronbach’s alpha = 0.74). The MDPS-M has a sensitivity of over 80% in detecting a high risk for mild depression in Japanese child-rearing women at the cutoff point of 9/10. Cronbach’s alpha for reliability in the sample of this study was 0.75.

*Japanese Resilience Scale (J-RS) *(Table 2)*.* This scale was developed in this study, and it is described in the following sections.

**Table 2:** Japanese resilience scale (J-RS) items

			Never	Rarely	Sometimes	Often	Always
**Joy**	**J-RS 1**	I am honestly pleased with my own success	□	□	□	□	□
	**J-RS 2**	I can forgive others even if they cause me to suffer	□	□	□	□	□
	**J-RS 3**	I can trust others even when they are dishonest	□	□	□	□	□
	**J-RS 4**	I can enjoy my daily life even when I have hard times	□	□	□	□	□
**Anger**	**J-RS 5**	It is easy to control my emotions in stressful situations	□	□	□	□	□
	**J-RS 6**	I am frustrated when things don’t go the way I had hoped	□	□	□	□	□
	**J-RS 7**	I can deal with difficult situations flexibly	□	□	□	□	□
	**J-RS 8**	I suddenly burst into rage when things are inconvenient for me	□	□	□	□	□
**Apprehension**	**J-RS 9**	I don’t keep on regretting something that is already over	□	□	□	□	□
	**J-RS 10**	I can move on even after a failure	□	□	□	□	□
	**J-RS 11**	When things go wrong, I take a pessimistic view of things	□	□	□	□	□
	**J-RS 12**	Even the smallest event can get me down	□	□	□	□	□
**Grief**	**J-RS 13**	I worry about what others think of me	□	□	□	□	□
	**J-RS 14**	I'm concerned about the little things that no one else seems to care about	□	□	□	□	□
	**J-RS 15**	Unsettled situations can lead me to become frustrated	□	□	□	□	□
	**J-RS 16**	Feeling alone when facing or working on difficult matters	□	□	□	□	□
**Willingness**	**J-RS 17**	I can overcome failures and take on new challenges	□	□	□	□	□
	**J-RS 18**	I can keep a positive mindset even when faced with difficult situations	□	□	□	□	□
	**J-RS 19**	Even if I fail, I can still follow my goals	□	□	□	□	□
	**J-RS 20**	I can believe in myself even when I feel anxious	□	□	□	□	□
**Social**	**J-RS 21**	I have someone to laugh and cry with	□	□	□	□	□
	**J-RS 22**	I have someone to share a good time with	□	□	□	□	□
	**J-RS 23**	I have someone who is staying by my side and taking care of me	□	□	□	□	□
	**J-RS 24**	I have someone who cares about me and gives me encouraging words	□	□	□	□	□
	**J-RS 25**	I have someone who is close to me and gives me tender care	□	□	□	□	□

*Abbreviations*: *J-RS* Japan Resilience Scale

The following is a list of the ways you might have felt. Please select how often you have felt this way recently

### Design and procedure

This study was conducted based on a cross-sectional research design. For the construction of the J-RS, candidate questions were developed through discussions among experts in internal medicine, psychiatry, and Kampo medicine. These questions were based on the theory of Kampo medicine, aiming to ensure they are easily understandable and acceptable to the Japanese population. The scale assesses how the subject has felt over the past month, and some of the items (J-RS 6, 8, 11, 12, 13, 14, 15, 16) are reverse scored. The final version of the J-RS contains 25 items across six subscales, all of which are rated on a 5-point scale ranging from “never true” to “always true” (Table 2). The total score ranges from 0 to 100, with higher scores reflecting greater resilience. The six subscales are composed of (i) Joy, (ii) Anger, (iii) Apprehension, (iv) Grief, (v) Willingness, and (vi) Social connection. Next, other assessment tools were selected to test the validity of the J-RS. Finally, mothers with children aged 0–5 years, who registered as volunteers at Kyoto University, were contacted by telephone. All questionnaires were sent by mail to those who agreed to participate, and they were asked to complete the questionnaire at home. Data were collected from 245 Japanese women between July 2021 and February 2022.

### Data analysis

To assess concurrent validity of the J-RS, correlation coefficients were calculated with a two-tailed test at 95% confidence and 80% power, with coefficients of 0.7 to 1 indicating strong correlation. With one explanatory variable, such as the resilience score, and 10 mental disorder cases, a minimum of 58 participants were needed, aligning with depression’s prevalence of 17.3% in Japan [34]. Finally, the analysis was conducted with 238 participants, excluding the seven who provided incomplete answers. To evaluate the construct validity of the J-RS, the correlations between the J-RS and other indicators were measured using Pearson’s correlation coefficient. Internal consistency (reliability) of the J-RS was assessed using Cronbach’s alpha coefficient. A confirmatory factor analysis (CFA) was conducted to assess a priori hypotheses about relations between observed variables and their underlying latent variables of the J-RS. To investigate the model’s goodness of fit, several fitness indicators were used: overall chi-squared, root mean square error of approximation (RMSEA), goodness of fit index (GFI), non-normed fit index (NNFI), Tucker Lewis index (TLI), normed fit index (NFI), comparative fit index (CFI), and the standardized root mean squared residual (SRMR). Model fit was evaluated using common fit indices. The model was considered to have an acceptable fit when RMSEA and SRMR were less than 0.08; GFI, NNFI, TLI, and NFI exceeded 0.80; and CFI exceeded 0.90. In addition, a χ2/df ratio below 5 was considered indicative of acceptable model fit [35]. After assessment of internal consistency (reliability) and construct validity of the J-RS, the predictive performance of the CES-D based on the J-RS total was evaluated using the areas under the curves (AUCs) of the receiver-operating characteristic (ROC) curves. Considering the prevailing circumstances in Japan, where postpartum mothers with depression are less inclined to seek consultation from specialists, greater emphasis was placed on sensitivity, thereby enabling screening by general practitioners. A clinically relevant cutoff point was determined to be the point at which sensitivity was greater than 80%, and sensitivity, specificity, positive predictive value (PPV), and negative predictive value (NPV) were calculated. Continuous variables are summarized as means ± standard deviation (SD), and categorical variables are presented as frequencies and percentages. R version 4.2.1 (R Foundation for Statistical Computing, Vienna, Austria) was used for the analyses. The CFA model was assessed with the R package “lavvan”. ROC curves were plotted with the R package “pROC”. A *p* value of < 0.05 was considered significant. All statistical analyses were performed by independent statisticians.

## Results

### Descriptive analysis and internal consistency of the items

The mean J-RS score in the present study was 61.3 ± 14.2. The score for each subscale of Joy, Anger, Apprehension, Grief, and Willingness was 9.9 ± 2.7, 7.7 ± 3.0, 7.8 ± 4.0, 7.2 ± 3.7, and 10.9 ± 2.9, respectively (range, 0–16). The Social connection subscale scored 17.7 ± 3.1 (range, 0–20) (Table 1b). The “Social connection” subscale had a higher score than the others. In addition, whereas the other five subscales related to emotional regulation followed a normal distribution, a tendency towards higher scores was observed in the “Social connection” subscale. The mean score of the CES-D was 10.2 ± 8.0, with 18.5% of participants showing depressive symptoms on the CES-D (≥ 16). All items were strongly correlated with the total score, and there was no increase in the overall reliability of the scale when any items were removed.

### Factor analysis for embodying the concept of the J-RS

CFA was conducted to test the factor structure of the J-RS. The sample consisted of 238 women who were rearing children. The CFA results indicated a good fit of the hypothesized six-factor model in relation to emotion and social connection to the data: χ^2^/df = 2.27, GFI = 0.831, NNFI = 0.885, TLI = 0.885, NFI = 0.837, CFI = 0.901, and SRMR = 0.066 (Table 3). The factor loadings for each item were as follows: Joy (0.49–0.76), Anger (0.51–0.82), Apprehension (0.76–0.83), Grief (0.63–0.78), Willingness (0.73–0.82), and Social connection (0.70–0.94) (Fig. 2A), indicating a moderate to strong relationship between the items and their respective factors. In the evaluation of the relationships between each item and factors based on the CFA, the contribution of “I have someone who stays by my side and takes care of me” (J-RS 23) and “I have someone who cares about me and gives me encouraging words” (J-RS 24) was especially high in the social aspect. These results provide support for the six-factor structure of the J-RS, which appears to be reliable and valid for emotions and social connection. Estimates of the measurement model, covariance, and variance, are shown in Suppl. Tables 1–3.

**Table 3:** Fit indices for the confirmatory factor analysis model

Model fit indices	χ^2^/df	RMSEA	GFI	NNFI	TLI	NFI	CFI	SRMR
Confirmatory factor analysis	2.27	0.073	0.831	0.885	0.885	0.837	0.901	0.066
Thresholds for acceptable fit	< 5	< 0.08	> 0.8	> 0.80	> 0.80	> 0.80	> 0.85	< 0.08
Thresholds for good fit	< 2	< 0.05	> 0.95	> 0.95	> 0.95	> 0.95	> 0.90	< 0.05

*Abbreviations*: *RMSEA* Root Mean Square Error of Approximation, *GFI* Goodness of Fit Index, *NNFI* Non-Normed Fit Index, *TLI* Tucker-Lewis Index, *NFI* Normed Fit Index, *CFI* Comparative Fit Index, *SRMR* Standardized Root Mean square Residual

### Reliability and validity of the J-RS

**Fig. 1:**
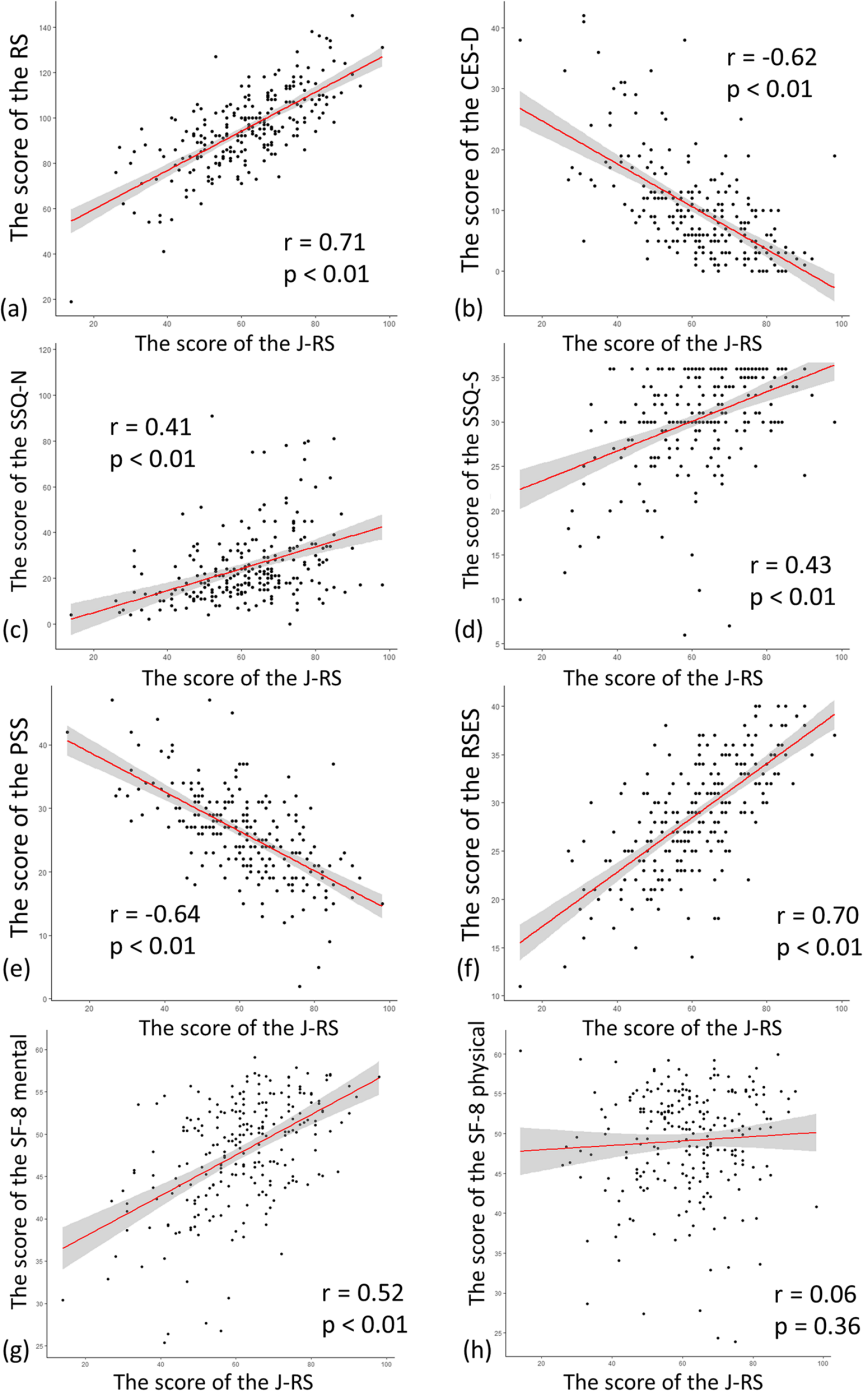
Correlations between the score of the J-RS and other measures. **a** A positive correlation with the RS. **b** A negative association with the CES-D. **c**, **d** the J-RS and the SSQ have a positive correlation with SSQ-number and SSQ-satisfaction. **e** A negative association with PSS, and (**f**) a positive correlation with RSES. **g** A positive relationship with SF- 8 mental. **h** No significant relationship with SF- 8 physical. J-RS, Japan Resilience Scale; RS, Resilience Scale; CES-D, Center for Epidemiologic Studies Depression; SSQ, Social Support Questionnaire; PSS, Perceived Stress Scale; RSES, Rosenberg Self-Esteem Scale

The overall Cronbach’s alpha coefficient for the J-RS was 0.92, and each subcategory ranged from 0.76 to 0.83 (Suppl. Table 4). The study examined the relationships between the J-RS and other measures including the RS, CES-D, RSES, SSQ, PSS, and SF- 8. The J-RS was positively correlated with the RS (*r* = 0.71, *p* < 0.01; Fig. 1a), indicating that the J-RS has validity. The RSES and the SSQ also showed positive correlations (RSES, *r* = 0.70, *p* < 0.01: Fig. 1f and SSQ Number *r* = 0.41, *p* < 0.01: Quantity *r* = 0.43, *p* < 0.01; Fig. 1c, d), which are consistent with previous concepts of resilience. In contrast, the J-RS was negatively correlated with the CES-D (*r* = − 0.62, *p* < 0.01; Fig. 1b), The J-RS was also negatively correlated with the PSS (*r* = − 0.64, *p* < 0.01; Fig. 1e). The J-RS showed a positive association with SF- 8 Mental (SF- 8 Mental *r* = 0.52, *p* < 0.01; SF- 8 Physical *r* = 0.06, *p* = 0.36, Fig. 1g, h). The J-RS also showed a negative association with MDPS-M (*r* = − 0.35, *p* < 0.01; Suppl. Figure 1) Moreover, the RS and J-RS showed similar correlations with the other scales in the present study; however, the J-RS showed stronger relationships than RS (Suppl. Table 5).

### Relationships between the six subscales of the J-RS and other indicators

The present study examined the extent to which the six subscales of the J-RS, namely five emotions (Joy, Anger, Apprehension, Grief, and Willingness), and Social connection are reflected in other indicators. The results of the bivariate correlations between each factor score and the total scores of other indicators are presented in Suppl. Table 6. All emotions were strongly reflected in the J-RS, with Pearson correlation coefficients as follows: Joy (0.72), Anger (0.73), Apprehension (0.83), Grief (0.75), Willingness (0.78), and Social connection (0.56). In contrast, the RS showed stronger correlations with Anger (0.62), Apprehension (0.63), and Willingness (0.73), but weaker correlations with Joy (0.45), Grief (0.42), and Social connection (0.29). These findings suggest that the evaluation items used in the RS differ from those used in the J-RS. In addition, the SSQ, a scale measuring social support, showed a higher contribution of the social factor than other factors. No significant correlations were found between the JR-S and SF- 8 Physical. SF- 8 Mental showed moderate correlations with Apprehension (0.48) and Grief (0.49), but weak correlations with Joy (0.28), Anger (0.34), Willingness (0.36), and Social connection (0.28).

### Detection of depressed subjects by the J-RS

**Fig. 2:**
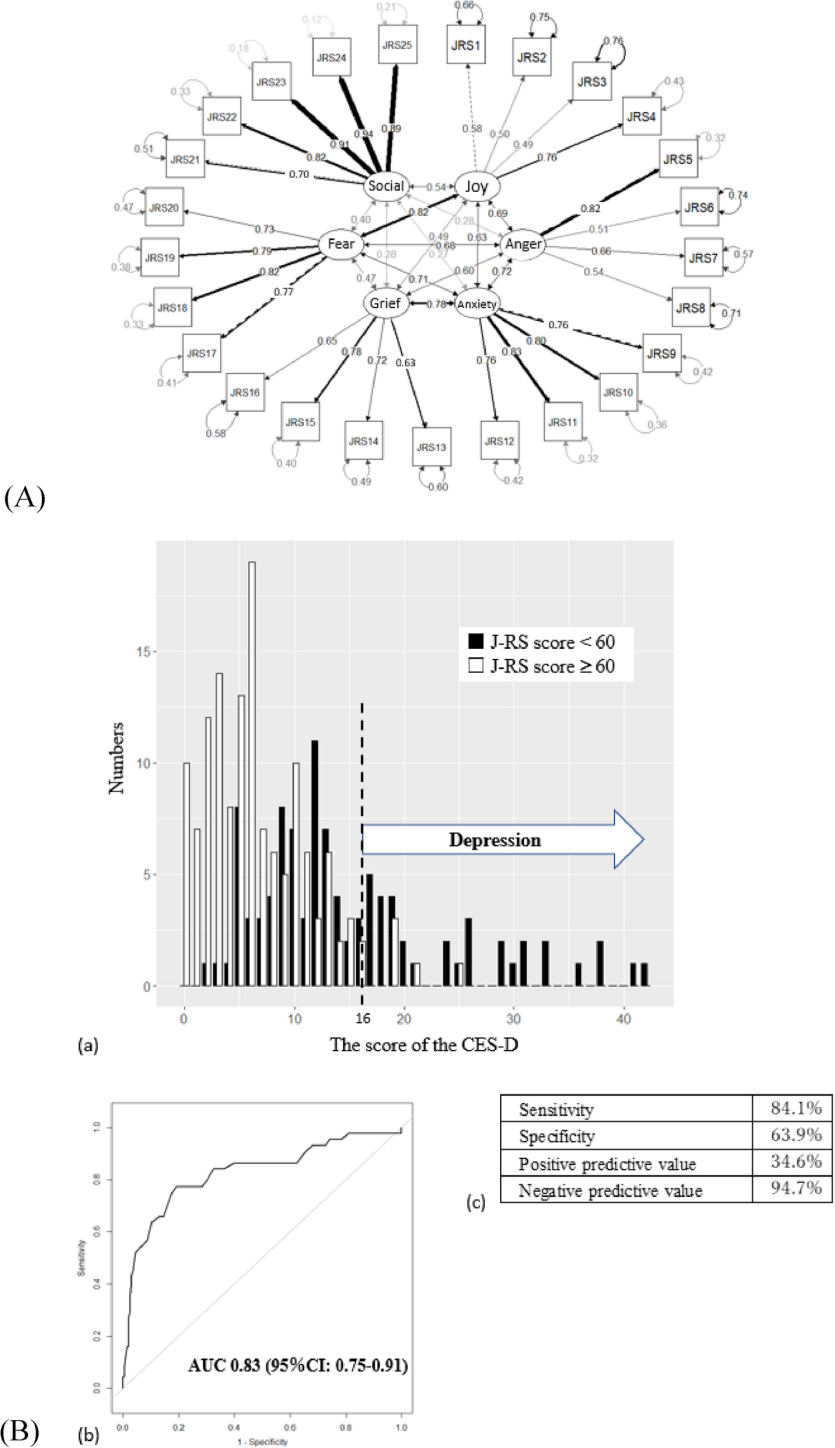
**A** Confirmatory factor analysis (CFA) for the components of the J-RS. The CFA results indicate a good fit of the hypothesized six-factor model related to emotional and social connection. CFA, confirmatory factor analysis; J-RS, Japan Resilience Scale. **B**. Score distribution, discrimination capability, and diagnostic accuracy of the J-RS. **a** Score distribution of the J-RS. **b** Discrimination capacity of the J-RS for the identification of depression based on the CES-D by ROC curves. **c** Sensitivity, specificity, positive predictive value, and negative predictive value for a score of 59 or less for a high risk for depression. J-RS, Japan Resilience Scale; CES-D, Center for Epidemiologic Studies Depression; ROC, receiver-operating characteristic

A lower score of the J-RS suggests a higher risk of depression, and the distribution of scores is depicted in Fig. 2B. Depression was assessed using the CES-D, which is commonly used to assess depressive symptoms. The J-RS score distributions varied between those with depressive symptoms (CES-D ≥ 16) and those without (CES-D < 16). The ROC curve of the J-RS is shown in Fig. 2B (AUC = 0.83, 95% CI: 0.75–0.91). The cut-off point was defined as the point at which the sensitivity was greater than 80%; the cutoff value of the J-RS was determined to be 59/60. J-RS’s sensitivity, specificity, PPV, and NPV were then calculated to be 84.1%, 63.9%, 4.6%, and 94.7%, respectively (Fig. 2B).

## Discussion

In the present study, a questionnaire was developed, and the reliability and validity of a new resilience scale were assessed. This scale considers cultural and social contexts of Japan, emphasizing emotional regulation, social bonds, and empathy among mothers raising children. The present study showed that the J-RS is strongly correlated with self-esteem and social support, which is quite consistent with the concept of resilience. The inverse correlation with perceived stress is also consistent with the original validation of the RS, indicating that resilience contains aspects of stress coping. In addition, the subjects in the present study were a suitable population for the assessment of resilience. Participants in the present study were from relatively affluent family backgrounds compared with national statistics [36]. However, the levels of self-esteem and perceived stress in this study were almost the same as the Japanese average [28, 32]. The percentage of participants with depressive symptoms on the CES-D is also consistent with the prevalence of depressed patients in the general population [34].

The J-RS exhibited a satisfactory level of internal consistency, with an overall Cronbach’s alpha coefficient of 0.92. Furthermore, each subcategory demonstrated acceptable reliability, with values ranging from 0.76 to 0.83, all of which fall within an acceptable range of reliability. Factor analysis of each question clearly showed that the J-RS is a valid measurement model consisting of six subscales, and each item in the questionnaire is strongly associated with its corresponding factor: the five emotions and social factors. In other words, the present results suggested new aspects of resilience: empathy, social connection, and emotional regulation may be components of resilience.

In the present study, the J-RS and resilience were strongly correlated in total scores, which is a stronger relationship than was present in the previous study [24]. Although not seen in the study of the Japanese version of the RS [24], the J-RS was also negatively correlated with perceived stress (*r* = − 0.64, *p* < 0.01; Fig. 1e), which is aligned with the concept of resilience. A low association was found between the RS and the social connectedness factor. This suggests that the components assessed by the J-RS may differ from those in conventional assessments of resilience. These findings imply that the low overall score of the RS in the Japanese validation study may be linked to Japan’s cultural and social background. Future studies examining the J-RS in.

 other countries with different cultural and social backgrounds may contribute to elucidating different components of resilience.

Emotional regulation is essential for maintaining mental health [37]. For instance, positive emotions contribute to the promotion of mental health [38]; however, excessive amplification might sometimes lead to negative outcomes [39]. Whereas chronic negative emotions have been associated with mental disorders [40], they also serve important functions, such as aiding in situational assessment and facilitating the avoidance of potential dangers [41]. Thus, appropriate regulation of all types of emotions is necessary [23]. The present scale focuses on emotional regulation as a key personal factor. Emotional regulation has also been reported to contribute not only to mental health, but also to physical health [23] and disease management [42]. For example, in the field of cardiovascular health, emotions such as “anger” and “hostility” have been identified as significant predictors of coronary heart disease (CHD) [43]. Conversely, positive emotions may play a protective role in the development of disease such as hypertension, diabetes mellitus, and respiratory tract infections [38]. Disease management is also associated with social connections, such as empathy and social support [44]. In addition, long-term studies have shown that emotions and social connections are also associated with the prognosis of patients with CHD [45]. Emotions are also known to affect social connections [46]. For example, social isolation, or a lack of social opportunity, gives rise to a sense of loneliness [47]. Loneliness, moreover, is closely related to resilience in Danish research [48].

Previous research on Japanese value priorities has highlighted the importance of social connection, ranking it as a highly valued trait in Japanese society [49]. The result that the “Social connection” subscale has a higher score than the others is likely affected by the Japanese cultural emphasis on collectivism and interpersonal harmony, where maintaining strong social ties is highly valued. The present findings also showed a positive correlation between the quantitative evaluation of social support and resilience, as well as a positive correlation between satisfaction with social support and resilience. In other words, having connections with society is one way to enhance resilience. This is likely a reflection of the Japanese cultural milieu. In addition, emotional regulation was also related to enhanced resilience [50], suggesting that the J-RS could be used as one of the indicators for treatment.

The results of the factor analysis indicated that the social connection category was strongly influenced by J-RS 23 and J-RS 24, emphasizing the importance of having someone present who can care for and provide encouragement to the individual. Previous resilience studies have typically examined the availability of support resources, such as the level of support from family and friends, without revealing the specific details [51]. The present study has contributed to a greater understanding of the social support resource.

In the present findings, the J-RS was negatively correlated with depressive symptoms. This means that people with higher resilience are less likely to develop depressive tendencies. The present findings also showed that the J-RS could assess depression with a sensitivity of 84.1% and specificity of 67.5%, using 59/60 as the cutoff point. Using the J-RS as a new screening tool, it would be possible to determine susceptibility to depression at an early stage. It is well known that depressed patients complain of physical symptoms [52]. We previously developed and validated a new scale, the MDPS-M [7], to detect depression based on physical symptoms. The MDPS-M is a unique and innovative scale to detect risk of depression without asking emotional questions. In the future, a combined evaluation of both scales may be effective.

SF- 8 is widely used to assess health-related QOL, including both mental and physical functioning. We analyzed the difference between health-related QOL and the J-RS in order to create a resilience scale as a tool for assessing mental health. In the present study, mental health-related QOL was associated with the J-RS and resilience, and it showed negative correlations with both depressive symptoms and perceived stress. However, the level of mental health-related QOL showed a weaker relationship with self-esteem than the J-RS and the RS (Suppl. Table 5). Mental health-related QOL reflected Apprehension and Grief well, whereas Joy, Anger, and Social connections were poorly related to health-related QOL (Suppl. Table 6). SF- 8 Mental was adequate in assessing mental QOL, but not for the dimension of resilience.

This study has several limitations that need to be addressed in future studies. First, participants in the present study were from relatively affluent family backgrounds compared with national statistics (21.1% affluent) [36]. However, the proportion of participants exhibiting depressive symptoms, as well as the mean scores on the level of self-esteem and perceived stress, were comparable to the Japanese average [28, 32]. Furthermore, no direct correlation was observed between income and resilience in the present study, suggesting that the sample is not necessarily distinct from the general population. Nonetheless, future studies should include participants with more diverse socioeconomic backgrounds to better understand the role of cultural and social factors in resilience. Second, this study used a cross-sectional design, meaning that data were collected at a single point in time. This design allows for the identification of associations between variables, but does not establish causal relationships, limiting the ability to determine the direction of effect. However, since a standardized screening process was implemented in this study, future longitudinal research would be beneficial to better predict long-term outcomes. Furthermore, a validation study is currently planned for a broader population (healthy individuals aged 20–60 years) to enhance the scale’s applicability across different age groups and sexes.

## Conclusions

The J-RS is a valid and reliable measure of resilience among Japanese mothers of child-rearing age. Developed to reflect Japanese cultural contexts, factor analysis confirmed the validity of each item, highlighting the emotional and social dimensions of resilience. Furthermore, comparisons with other resilience scales suggest that the conceptual components of resilience may vary across cultural backgrounds.

## Supplementary Information

Supplementary Material 1.

Supplementary Material 2.

Supplementary Material 3.

Supplementary Material 4.

Supplementary Material 5.

Supplementary Material 6.

Supplementary Material 7.

## Data Availability

The data that support the findings of this study are available upon reasonable request from the corresponding author, K.H. Access to the data is limited to researchers affiliated with academic or medical institutions, subject to approval by the institutional ethics committee. The data are not publicly available due to their containing information that could compromise the privacy of research participants.

## Abbreviations

CES-D: Center for Epidemiologic Studies Depression
J-RS: Japan Resilience Scale
MDPS-M: Multidimensional Physical Scale for Mothers
PSS: Perceived Stress Scale
QOL: Quality of life
RS: Resilience Scale
RSES: Rosenberg Self-Esteem Scale
SF- 8: Short Form Health Survey
SSQ: Social Support Questionnaire
