# Supplementary figures and images for: The validity of a new resilience scale: the Japan Resilience Scale (J-RS) for mothers with a focus on cultural aspects
Source: BMC Public Health. 2025 Apr 28;25:1569. doi: 10.1186/s12889-025-22765-6 (PMC12036222; doi:10.1186/s12889-025-22765-6)

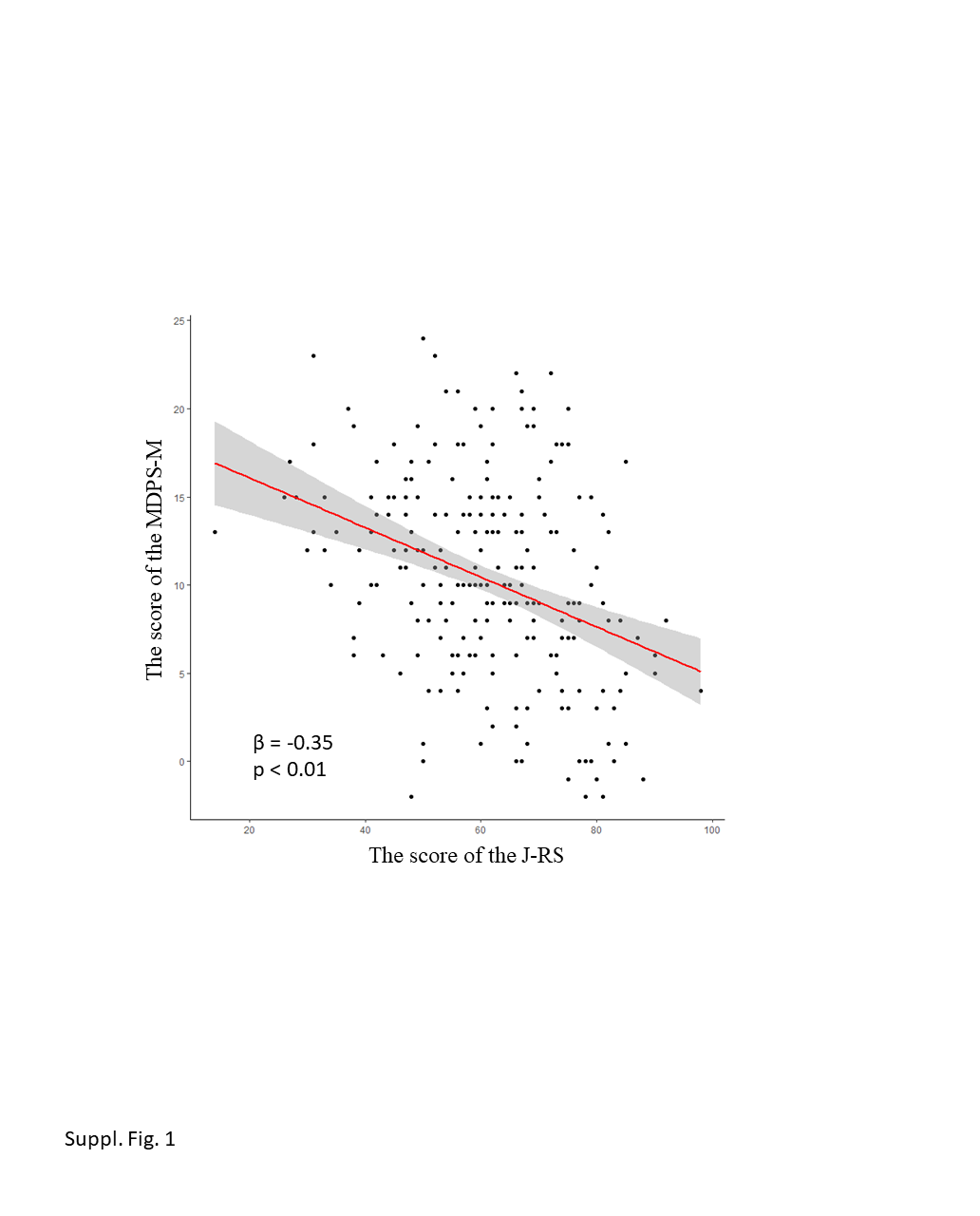

Supplement: Supplementary file 1 — Supplementary Material 1. [file 12889_2025_22765_MOESM1_ESM.tif]
