# Supplementary material for: The validity of a new resilience scale: the Japan Resilience Scale (J-RS) for mothers with a focus on cultural aspects
Source: BMC Public Health. 2025 Apr 28;25:1569. doi: 10.1186/s12889-025-22765-6 (PMC12036222; doi:10.1186/s12889-025-22765-6)
Supplement: Supplementary file 2 — Supplementary Material 2. [file 12889_2025_22765_MOESM2_ESM.docx]

**Suppl. Table 1. Estimates of the measurement model**

Abbreviations: J-RS, Japan Resilience Scale

| **Latent Variables:** |  |  |  |  |  |  |
| --- | --- | --- | --- | --- | --- | --- |
|  | **Estimate** | **Std.Err** | **z-value** | **P(>\|z\|)** | **Std.lv** | **Std.all** |
| **Joy**$\boldsymbol{\cong}$ |  |  |  |  |  |  |
| J-RS1 | 1.000 |  |  |  | 0.437 | 0.585 |
| J-RS2 | 1.112 | 0.179 | 6.220 | 0.000 | 0.486 | 0.500 |
| J-RS3 | 1.197 | 0.196 | 6.116 | 0.000 | 0.523 | 0.490 |
| J-RS4 | 1.696 | 0.206 | 8.225 | 0.000 | 0.742 | 0.758 |
| **Anger**$\boldsymbol{\cong}$ |  |  |  |  |  |  |
| J-RS5 | 1.000 |  |  |  | 0.844 | 0.825 |
| J-RS6 | 0.526 | 0.072 | 7.343 | 0.000 | 0.444 | 0.506 |
| J-RS7 | 0.724 | 0.075 | 9.677 | 0.000 | 0.611 | 0.660 |
| J-RS8 | 0.748 | 0.096 | 7.816 | 0.000 | 0.631 | 0.537 |
| **Apprehension**$\boldsymbol{\cong}$ |  |  |  |  |  |  |
| J-RS9 | 1.000 |  |  |  | 0.944 | 0.760 |
| J-RS10 | 0.985 | 0.079 | 12.507 | 0.000 | 0.930 | 0.799 |
| J-RS11 | 0.968 | 0.075 | 12.984 | 0.000 | 0.914 | 0.827 |
| J-RS12 | 0.946 | 0.080 | 11.836 | 0.000 | 0.892 | 0.760 |
| **Grief**$\boldsymbol{\cong}$ |  |  |  |  |  |  |
| J-RS13 | 1.000 |  |  |  | 0.696 | 0.631 |
| J-RS14 | 1.229 | 0.142 | 8.684 | 0.000 | 0.855 | 0.715 |
| J-RS15 | 1.336 | 0.146 | 9.141 | 0.000 | 0.929 | 0.775 |
| J-RS16 | 1.147 | 0.142 | 8.057 | 0.000 | 0.798 | 0.646 |
| **Willingness**$\boldsymbol{\cong}$ |  |  |  |  |  |  |
| J-RS17 | 1.000 |  |  |  | 0.644 | 0.767 |
| J-RS18 | 1.159 | 0.091 | 12.769 | 0.000 | 0.747 | 0.817 |
| J-RS19 | 1.015 | 0.082 | 12.316 | 0.000 | 0.654 | 0.790 |
| J-RS20 | 1.019 | 0.090 | 11.313 | 0.000 | 0.657 | 0.731 |
| **Social**$\boldsymbol{\cong}$ |  |  |  |  |  |  |
| J-RS21 | 1.000 |  |  |  | 0.567 | 0.697 |
| J-RS22 | 0.932 | 0.077 | 12.050 | 0.000 | 0.528 | 0.820 |
| J-RS23 | 1.054 | 0.080 | 13.256 | 0.000 | 0.598 | 0.907 |
| J-RS24 | 1.117 | 0.082 | 13.662 | 0.000 | 0.633 | 0.939 |
| J-RS25 | 1.049 | 0.081 | 13.029 | 0.000 | 0.595 | 0.890 |
