# Supplementary material for: The validity of a new resilience scale: the Japan Resilience Scale (J-RS) for mothers with a focus on cultural aspects
Source: BMC Public Health. 2025 Apr 28;25:1569. doi: 10.1186/s12889-025-22765-6 (PMC12036222; doi:10.1186/s12889-025-22765-6)
Supplement: Supplementary file 3 — Supplementary Material 3. [file 12889_2025_22765_MOESM3_ESM.docx]

**Suppl. Table 2. Estimates of covariance**

Abbreviations: J-RS, Japan Resilience Scale

| **Covariances:** | | | | | | |
| --- | --- | --- | --- | --- | --- | --- |
|  | **Estimate** | **Std.Err** | **z-value** | **P(>\|z\|)** | **Std.lv** | **Std.all** |
| **Joy**$\boldsymbol{\approx}$ | | | | | | |
| Anger | 0.254 | 0.044 | 5.821 | 0.000 | 0.688 | 0.688 |
| Apprehension | 0.259 | 0.047 | 5.553 | 0.000 | 0.629 | 0.629 |
| Grief | 0.149 | 0.033 | 4.469 | 0.000 | 0.488 | 0.488 |
| Willingness | 0.230 | 0.037 | 6.275 | 0.000 | 0.818 | 0.818 |
| Social | 0.134 | 0.026 | 5.121 | 0.000 | 0.540 | 0.540 |
| **Anger**$\boldsymbol{\approx}$ | | | | | | |
| Apprehension | 0.577 | 0.082 | 7.068 | 0.000 | 0.725 | 0.725 |
| Grief | 0.353 | 0.062 | 5.705 | 0.000 | 0.601 | 0.601 |
| Willingness | 0.372 | 0.054 | 6.825 | 0.000 | 0.683 | 0.683 |
| Social | 0.134 | 0.038 | 3.500 | 0.000 | 0.280 | 0.280 |
| **Apprehension**$\boldsymbol{\approx}$ | | | | | | |
| Grief | 0.515 | 0.079 | 6.507 | 0.000 | 0.785 | 0.785 |
| Willingness | 0.429 | 0.062 | 6.892 | 0.000 | 0.705 | 0.705 |
| Social | 0.147 | 0.041 | 3.551 | 0.000 | 0.274 | 0.274 |
| **Grief**$\boldsymbol{\approx}$ | | | | | | |
| Willingness | 0.210 | 0.043 | 4.908 | 0.000 | 0.468 | 0.468 |
| Social | 0.112 | 0.032 | 3.436 | 0.001 | 0.283 | 0.283 |
| **Willingness**$\boldsymbol{\approx}$ | | | | | | |
| Social | 0.146 | 0.030 | 4.797 | 0.000 | 0.400 | 0.400 |
