# Supplementary material for: The validity of a new resilience scale: the Japan Resilience Scale (J-RS) for mothers with a focus on cultural aspects
Source: BMC Public Health. 2025 Apr 28;25:1569. doi: 10.1186/s12889-025-22765-6 (PMC12036222; doi:10.1186/s12889-025-22765-6)
Supplement: Supplementary file 4 — Supplementary Material 4. [file 12889_2025_22765_MOESM4_ESM.docx]

**Suppl. Table 3. Estimates of variance**

Abbreviations: J-RS, Japan Resilience Scale

| **Variances:** | | | | | | |
| --- | --- | --- | --- | --- | --- | --- |
|  | **Estimate** | **Std.Err** | **z-value** | **P(>\|z\|)** | **Std.lv** | **Std.all** |
| **J-RS1** | 0.368 | 0.038 | 9.677 | 0.000 | 0.368 | 0.658 |
| **J-RS2** | 0.709 | 0.070 | 10.132 | 0.000 | 0.709 | 0.750 |
| **J-RS3** | 0.869 | 0.085 | 10.176 | 0.000 | 0.869 | 0.760 |
| **J-RS4** | 0.407 | 0.055 | 7.339 | 0.000 | 0.407 | 0.425 |
| **J-RS5** | 0.335 | 0.056 | 5.983 | 0.000 | 0.335 | 0.320 |
| **J-RS6** | 0.572 | 0.056 | 10.142 | 0.000 | 0.572 | 0.744 |
| **J-RS7** | 0.485 | 0.053 | 9.146 | 0.000 | 0.485 | 0.565 |
| **J-RS8** | 0.980 | 0.098 | 10.003 | 0.000 | 0.980 | 0.711 |
| **J-RS9** | 0.653 | 0.071 | 9.212 | 0.000 | 0.653 | 0.423 |
| **J-RS10** | 0.490 | 0.056 | 8.698 | 0.000 | 0.490 | 0.362 |
| **J-RS11** | 0.386 | 0.047 | 8.189 | 0.000 | 0.386 | 0.316 |
| **J-RS12** | 0.582 | 0.063 | 9.205 | 0.000 | 0.582 | 0.422 |
| **J-RS13** | 0.731 | 0.077 | 9.496 | 0.000 | 0.731 | 0.602 |
| **J-RS14** | 0.697 | 0.081 | 8.644 | 0.000 | 0.697 | 0.488 |
| **J-RS15** | 0.573 | 0.075 | 7.634 | 0.000 | 0.573 | 0.399 |
| **J-RS16** | 0.891 | 0.095 | 9.382 | 0.000 | 0.891 | 0.583 |
| **J-RS17** | 0.291 | 0.033 | 8.920 | 0.000 | 0.291 | 0.412 |
| **J-RS18** | 0.278 | 0.034 | 8.092 | 0.000 | 0.278 | 0.333 |
| **J-RS19** | 0.258 | 0.030 | 8.587 | 0.000 | 0.258 | 0.376 |
| **J-RS20** | 0.375 | 0.040 | 9.312 | 0.000 | 0.375 | 0.465 |
| **J-RS21** | 0.340 | 0.033 | 10.366 | 0.000 | 0.340 | 0.514 |
| **J-RS23** | 0.077 | 0.009 | 8.087 | 0.000 | 0.077 | 0.177 |
| **J-RS24** | 0.054 | 0.008 | 6.447 | 0.000 | 0.054 | 0.118 |
| **J-RS25** | 0.092 | 0.011 | 8.618 | 0.000 | 0.092 | 0.207 |
| **Joy** | 0.191 | 0.042 | 4.507 | 0.000 | 1.000 | 1.000 |
| **Anger** | 0.712 | 0.102 | 6.960 | 0.000 | 1.000 | 1.000 |
| **Apprehension** | 0.891 | 0.134 | 6.659 | 0.000 | 1.000 | 1.000 |
| **Grief** | 0.484 | 0.097 | 5.005 | 0.000 | 1.000 | 1.000 |
| **Willingness** | 0.415 | 0.062 | 6.704 | 0.000 | 1.000 | 1.000 |
| **Social** | 0.321 | 0.053 | 6.065 | 0.000 | 1.000 | 1.000 |
