# Supplementary material for: The validity of a new resilience scale: the Japan Resilience Scale (J-RS) for mothers with a focus on cultural aspects
Source: BMC Public Health. 2025 Apr 28;25:1569. doi: 10.1186/s12889-025-22765-6 (PMC12036222; doi:10.1186/s12889-025-22765-6)
Supplement: Supplementary file 5 — Supplementary Material 5. [file 12889_2025_22765_MOESM5_ESM.docx]

**Suppl. Table 4. Cronbach’s alpha coefficient for the J-RS**

| **Dimension** | **The number of items** | **Internal consistency reliability**  **(Cronbach’s alpha coefficient)** |
| --- | --- | --- |
| J-RS Joy | 4 | 0.79 |
| J-RS Anger | 4 | 0.79 |
| J-RS Apprehension | 4 | 0.76 |
| J-RS Grief | 4 | 0.79 |
| J-RS Willingness | 4 | 0.78 |
| J-RS Social connections | 5 | 0.83 |
| J-RS total | 25 | 0.92 |
