# Supplementary material for: The validity of a new resilience scale: the Japan Resilience Scale (J-RS) for mothers with a focus on cultural aspects
Source: BMC Public Health. 2025 Apr 28;25:1569. doi: 10.1186/s12889-025-22765-6 (PMC12036222; doi:10.1186/s12889-025-22765-6)
Supplement: Supplementary file 6 — Supplementary Material 6. [file 12889_2025_22765_MOESM6_ESM.docx]

**Suppl. Table 5. Coefficients among the J-RS, the RS and other measures**

Abbreviations: J-RS, Japan Resilience Scale; RS, Resilience Scale; SD, standard deviation; CES-D, Center for Epidemiologic Studies Depression RSES, Rosenberg Self-Esteem Scale; PSS, Perceived Stress Scale; MDPS-M, Multidimensional Physical Scale for mothers; SSQ, Social Support Questionnaire;

|  | J-RS | | RS | | SF-8 Physical | | SF-8 Mental | |
| --- | --- | --- | --- | --- | --- | --- | --- | --- |
| (N=238) | r | p value | r | p value | r | p value | r | p value |
| CES-D | -0.62 | < 0.01 | -0.56 | < 0.01 | -0.22 | 0.0007 | -0.59 | < 0.01 |
| RSES | 0.70 | < 0.01 | 0.69 | < 0.01 | -0.01 | 0.85 | 0.47 | < 0.01 |
| PSS | -0.64 | < 0.01 | -0.57 | < 0.01 | -0.17 | 0.01 | -0.64 | < 0.01 |
| MDPS-M | -0.35 | < 0.01 | -0.24 | < 0.01 | -0.33 | < 0.01 | -0.31 | < 0.01 |
| SSQ Number | 0.41 | < 0.01 | 0.35 | < 0.01 | 0.09 | 0.19 | 0.28 | < 0.01 |
| SSQ Satisfaction | 0.43 | < 0.01 | 0.28 | < 0.01 | -0.01 | 0.91 | 0.36 | < 0.01 |
| SF-8 Physical | 0.06 | 0.362 | 0.03 | 0.661 |  | | | |
| SF-8 Mental | 0.52 | < 0.01 | 0.43 | < 0.01 |  |  |  |  |
