# Supplementary material for: The validity of a new resilience scale: the Japan Resilience Scale (J-RS) for mothers with a focus on cultural aspects
Source: BMC Public Health. 2025 Apr 28;25:1569. doi: 10.1186/s12889-025-22765-6 (PMC12036222; doi:10.1186/s12889-025-22765-6)
Supplement: Supplementary file 7 — Supplementary Material 7. [file 12889_2025_22765_MOESM7_ESM.docx]

**Suppl. Table 6.** **Relationship between six subscales of the J-RS and other indicators**

|  |  | **J-RS Total** | **RS** | **CES-D** | **RSES** | **PSS** | **MDPS-M** | **SSQ-N** | **SSQ-S** | **SF-8 Physical** | **SF-8 Mental** |
| --- | --- | --- | --- | --- | --- | --- | --- | --- | --- | --- | --- |
| r **(**$\boldsymbol{R}^{\boldsymbol{2}}$**)** | | | | | | | | | | | |
| J-RS Total | |  | 0.71^*^  (0.51) | -0.62^*^  (0.38) | 0.70^*^  (0.48) | -0.64^*^  (0.41) | -0.35^*^  (0.13) | 0.41^*^  (0.17) | 0.43^*^  (0.19) | 0.06  (0.004) | 0.52^*^  (0.27) |
| J-RS | Joy | 0.72^*^  (0.51) | 0.45^*^  (0.20) | -0.39^*^  (0.15) | 0.41^*^  (0.19) | -0.40^*^  (0.17) | -0.27^*^  (0.07) | 0.21^*^  (0.05) | 0.38^*^  (0.15) | 0.09  (0.01) | 0.28^*^  (0.08) |
|  | Anger | 0.73^*^  (0.53) | 0.62^*^  (0.38) | -0.38^*^  (0.15) | 0.45^*^  (0.19) | -0.52^*^  (0.26) | -0.21^*^  (0.05) | 0.23^*^  (0.05) | 0.21^+^  (0.04) | -0.04  (0.001) | 0.34^*^  (0.11) |
|  | Apprehension | 0.83^*^  (0.69) | 0.63^*^  (0.39) | -0.50^*^  (0.25) | 0.63^*^  (0.39) | -0.57^*^  (0.32) | -0.30^*^  (0.09) | 0.34^*^  (0.12) | 0.31^*^  (0.10) | 0.03  (0.001) | 0.48^*^  (0.23) |
|  | Grief | 0.75^*^  (0.57) | 0.42^*^  (0.18) | -0.45^*^  (0.21) | 0.54^*^  (0.27) | -0.49^*^  (0.23) | -0.36^*^  (0.13) | 0.29^*^  (0.08) | 0.21^+^  (0.05) | 0.14^+^  (0.02) | 0.49^*^  (0.24) |
|  | Willingness | 0.78^*^  (0.61) | 0.73^*^  (0.53) | -0.56^*^  (0.31) | 0.70^*^  (0.50) | -0.51^*^  (0.27) | -0.16^+^  (0.02) | 0.33^*^  (0.11) | 0.35^*^  (0.12) | 0.03  (0.001) | 0.36^*^  (0.13) |
|  | Social | 0.56^*^  (0.31) | 0.29^*^  (0.08) | -0.42^*^  (0.18) | 0.32^*^  (0.09) | -0.31^*^  (0.09) | -0.23^*^  (0.05) | 0.37^*^  (0.13) | 0.46^*^  (0.21) | 0.01  (0.0002) | 0.28^*^  (0.08) |

Abbreviations: J-RS, Japan Resilience Scale; RS, Resilience Scale; SD, standard deviation; CES-D, Center for Epidemiologic Studies Depression; SSQ, Social Support Questionnaire; PSS, Perceived Stress Scale; RSES, Rosenberg Self-Esteem Scale
